# A System Based on Photoplethysmography and Photobiomodulation for Autonomic Nervous System Measurement and Adjustment

**DOI:** 10.3390/life13020564

**Published:** 2023-02-17

**Authors:** Yi-Chia Shan, Wei Fang, Jih-Huah Wu

**Affiliations:** 1 Department of Information and Telecommunications Engineering, Ming Chuan University, No. 5, Deming Rd., Gweishan Township, Taoyuan 333, Taiwan; 2 Department of Biomechatronics Engineering, National Taiwan University, No. 1, Sec. 4, Roosevelt Rd., Taipei 106, Taiwan; 3 Department of Biomedical Engineering, Ming Chuan University, No. 5, Deming Rd., Gweishan Township, Taoyuan 333, Taiwan

**Keywords:** autonomic nervous system, heart rate variability, photoplethysmography, photobiomodulation

## Abstract

(1) Background: The imbalance of the autonomic nervous system (ANS) is common worldwide. Many people have high tension when the sympathetic nervous system is hyperactive or low attention when the parasympathetic nervous system is hyperactive. To improve autonomic imbalance, a feasible and integrated system was proposed to measure and affect the ANS status. (2) Methods: The proposed system consists of a signal-processing module, an LED stimulation module, a photoplethysmography (PPG) sensor and an LCD display. The heart rate variability (HRV) and ANS status can be analyzed from PPG data. To confirm HRV analysis from PPG data, an electrocardiogram (ECG) device was also used to measure HRV. Additionally, photobiomodulation (PBM) was used to affect the ANS status, and two acupuncture points (Neiguan (PC6) and Shenmen (HT7)) were stimulated with different frequencies (10 Hz and 40 Hz) of PBM. (3) Results: Two subjects were tested with the developed system. HRV metrics were discussed in the time domain and frequency domain. HRV metrics have a similar change trend on PPG and ECG signals. In addition, the SDNN was increased, and the parasympathetic nervous system (PNS: HF (%)) was enhanced with a 10 Hz pulse rate stimulation at the Neiguan acupoint (PC6). Furthermore, the SDNN was increased, and the sympathetic nervous system (SNS: LF (%)) was enhanced with a 40 Hz pulse rate stimulation at the Shenmen (HT7) acupoint. (4) Conclusion: A prototype to measure and affect the ANS was proposed, and the functions were feasible. The test results show that stimulating the Neiguan (PC6) acupoint can inhibit the SNS. In contrast, stimulating the Shenmen (HT7) acupoint can activate the SNS. However, more experiments must be conducted to confirm the effect by choosing different pulse rates, dosages and acupoints.

## 1. Introduction

The ANS includes two major branches. One is the sympathetic system, associated with the mobilization. Another is the parasympathetic system, associated with vegetative and restorative functions. Normally, the activity of these branches is in dynamic balance. Autonomic imbalance is when one branch of the ANS dominates over the other and is associated with a lack of dynamic flexibility and health [1]. The prevalence of autonomic imbalances is increasing worldwide. Many people encounter high competition and fast-paced life in modern society, and therefore they may keep in the state of high tension or low attention. HRV may be used to assess autonomic imbalances, diseases and mortality. Parasympathetic activity and HRV have been associated with various conditions, including cardiovascular disease [1]. In addition, HRV has been used as an indicator of SNS and PNS activity, as well as autonomic nervous activity [2,3]. The heartbeat rhythm is regulated by the sinoatrial node (pacemaker). The rhythm can be affected by the ANS and the cardiac output for body requirements [4]. The definitions of HRV terms and the standard measurement methods have been developed and specified [2,3]. HRV can be used to assess cardiovascular disease and psychological status. For example, the changes in HRV have been associated with severe cardiovascular, metabolic and mental disorders [2,5]. HRV measurement is approved to be an effective method for detecting early cardiovascular disease [6]. HRV can be used as a risk judgment factor of coronary artery occlusion, to predict coronary occlusion situations [7]. HRV is impacted by stress and is used to assess psychological health and stress objectively [8].

PPG is an optical technique that can be used to detect blood volume changes in the microvascular of tissue. There are many commercial medical devices for measuring oxygen saturation, blood pressure, cardiac output and autonomic function and detecting peripheral vascular disease [9,10]. PPG has the advantages of simplicity, non-invasiveness, small size and low cost. Compared to ECG, there is a high degree of correlation in the temporal domain, frequency domain and in nonlinear dynamic analyses between HRV measures derived from PPG and ECG [11]. PPG is widely used in smartphones and smart watches. The validation of the Apple Watch for HRV measurements during relaxation and mental stress in healthy subjects was confirmed [12].

Photobiomodulation (PBM) has been applied to the medical field for many years [13,14]. A low-level laser can help to improve postoperative care in peripheral nerve surgery [15], reduce the pain of chronic disease [16] and increase the wound tensile strength [17]. In addition, an LED has a similar effect compared with laser, such as wound healing, pain relief and angiogenesis [18]. In our previous study, a single laser was used to radiate the Neiguan (PC6) acupoint, and the parasympathetic nervous system (PNS) and heart rate variability (HRV) can be affected [19]. A low-level laser array was used to stimulate the palm of the subjects under eyes open and closed conditions, and such stimulation caused significant changes in the intensity of the subject’s brain waves [20,21]. A higher dosage LED array can induce more significant intensity changes in brain waves than the laser in lower dosage [22]. Thus, laser or LED acupuncture can be called light acupuncture (LA).

To improve the ANS imbalances and HRV metrics in real-time, an integrated system that can immediately measure and affect the ANS status is needed. This study aimed to develop a real-time ANS monitoring and influencing system based on PPG and LA. PPG is used to obtain ANS information, and LA is for PBM. For example, the user can use PBM with different pulse rates on specific acupoints to affect the ANS status. The reference criteria (not a standard) are in Table 1.

## 2. Materials and Methods

### 2.1. The Proposed System

**Figure 1:**
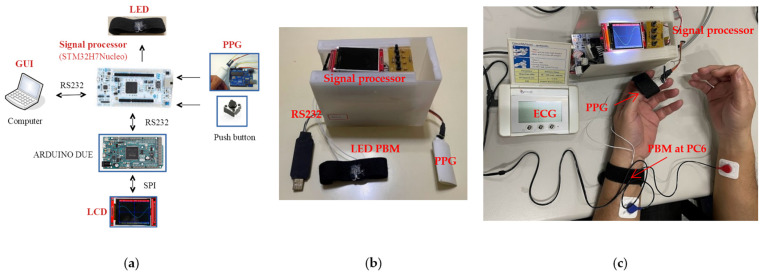
The illustration of the proposed system. (**a**) The prototype includes the signal processor, PPG sensor, LED stimulation module, and LCD display. (**b**) The final version of the prototype. (**c**) The experiment photo.

The proposed techniques and the system implementation are described as follows. The prototype includes a signal-processing module, a PPG sensor, a LED biostimulation module, and an LCD display. Figure 1a is the prototype of the system, and Figure 1b is the final version of the prototype. The experimental photo is shown in Figure 1c. The heart rate can be measured by the PPG sensor, and the measured position was left thumb. The PPG signal was sent to the signal-processing module, then the analog signal was converted to the digital signal through the internal analog digital conversion (ADC), and the sampling rate is 250 points/s. The signal-processing module can calculate the HRV metrics in the time domain and frequency domain from the PPG signal, and the related data can be stored in Flash RAM and shown on the LCD screen through Arduino processor. In addition, the user can press the button to activate the LED module to perform LA. The developed system is described in Section 2.1.1, Section 2.1.2, Section 2.1.3 and Section 2.1.4.

#### 2.1.1. Signal-Processing Module

The signal-processing module is the commercially available microprocessor STM32H743 Nucleo board, and the main functions are PPG data collection, HRV calculation, LED driven and RS232 communication.

#### 2.1.2. PPG Sensor

The systolic pressure and diastolic pressure correspond to the variation in the heartbeat, and the information on the heartbeat changes can be obtained by measuring the reflected light variability of the blood vessel. The PPG sensor is KSRobot KSM014, and the measured position is recommended to be placed on the left thumb. The other fingers can also be measured, but the signal is more manifested on the thumb than other fingers.

#### 2.1.3. LED Biostimulation Module

An infrared LED (B1591IRP-20C-000252) with a center wavelength of 850 nm was setup in the LED biostimulation module. The output power of LED is 20 mW, and the energy density is 20 J/cm^2^. The signal-processing module drives the LED module with the pulse width modulation (PWM) and the pulse rate is adjustable. In the test, we set two kinds of pulse rates, 10 Hz and 40 Hz, and the duty cycle was 50%.

#### 2.1.4. LCD Display

A 2.2-inch LCD display (QDtech_2.2) with a resolution of 176 × 220 was set up which can be controlled through Serial Peripheral Interface (SPI).

### 2.2. ECG HRV Measurement Device

HRV was also measured with an ECG medical device (CheckMyHeart; DailyCare BioMedical, Inc., Zhongli City, Taiwan).

### 2.3. Data Processing

#### 2.3.1. HRV Metric Calculation

HRV metrics are generally divided into two categories, time domain and frequency domain analysis. Regarding the time domain analysis, the five-minute time (short-term measurement) is used, such as the mean of the intervals, the standard deviation of the intervals (SDNN), the percentage of adjacent intervals that differ more than 50 ms (pNN50) and the root mean square of successive differences between heartbeats (RMSSD). These HRV metrics are not interchangeable and do not necessarily reflect similar physiology. For example, the SDNN is related to the total power (variance), whereas both RMSSD and pNN50 detect high-frequency oscillations [23]. In the frequency domain analysis, the Fourier transform is used to transfer the heartbeat information (a sequence of R-R intervals) to the spectrum and provide information on how power (variance) is distributed as a function of frequency [23]. The high-frequency (HF) range is from 0.15 to 0.4 Hz, which can be regarded as an indicator of parasympathetic activity. The low -frequency (LF) range is from 0.04 to 0.15 Hz, which can be regarded as an index of joint control between sympathetic and parasympathetic nerves. The very low-frequency (VLF) range is below 0.04 Hz, which can indicate sympathetic activity [2]. VLF was not discussed in this study since short-term records were used. The LF and HF powers are usually reported in normalized (relative or fractional) units, representing the relative value of each power in proportion to the total power. This is carried out to minimize the impact of changes in total power on the value of HF and LF components [23]. The relevant indices and signals are defined as follows [19]:

HF (%): the frequency band from 0.15–0.4 Hz, shown as a percentage.

$$\mathrm{HF}\;\left(\%\right)=\frac{\mathrm{HF}\;\mathrm{power}}{\left(\mathrm{HF}\;\mathrm{power}+\mathrm{LF}\;\mathrm{power}\right)}$$

LF (%): the frequency band from 0.04–0.15 Hz, shown as a percentage.

$$\mathrm{LF}\;\left(\%\right)=\frac{\mathrm{LF}\;\mathrm{power}}{\left(\mathrm{HF}\;\mathrm{power}+\mathrm{LF}\;\mathrm{power}\right)}$$

LF:HF ratio: the ratio of LF power to HF power.

$$\mathrm{LF}:\mathrm{HF}=\frac{\mathrm{LF}\;\mathrm{power}}{\mathrm{HF}\;\mathrm{power}}$$

SDNN (ms): the standard deviation of the R-R intervals seen on the ECG and the standard deviation of the P-P intervals seen from the PPG.

Since there are no comprehensive investigations of all indices in a large number of normal populations, there are some test results from a small number of subjects that can be referred to, shown in Table 1 [2]. In addition, SDNN values can predict both morbidity and mortality. Based on 24 h monitoring of the patients after acute myocardial infarction (AMI), the estimated mortality rate of SDNN values below 50 ms is 33%, 50–100 ms is 16% and greater than 100 ms is 9% [24].

#### 2.3.2. Data Agreements and Comparison of HRV Metrics

For the analysis of the agreement of HRV metrics between PPG and ECG, there are some methods to determine the agreement [25]. The Pearson correlation coefficient (PCC) is a common method to observe the linear correlation between two data sets. The strength of relationship can be anywhere between −1 and +1. The stronger the correlation, the closer the correlation coefficient comes to ±1. A correlation coefficient of zero indicates that no linear relationship exists between two continuous variables. The Pearson correlation coefficient higher than 0.90 represents a very high positive (negative) correlation; lower than 0.90 and higher than 0.70 is a high positive (negative) correlation [26].

Lin’s Concordance Correlation Coefficient (LCCC) is used to assess the degree of equivalence between a new laboratory method and a golden standard method. The criteria are that there is almost perfect agreement if LCCC > 0.99, substantial agreement if 0.95 < LCCC < 0.99, moderate agreement if 0.90 < LCCC < 0.95 and poor agreement if LCCC < 0.90 [27].

Another frequently applied technique to assess the agreement between two methods is the Bland–Altman analysis [28]. The Bland–Altman ratio (BA ratio) was calculated by dividing half the range of the limits of agreement (LoA) by the mean of the pair of means of each HRV metric. The criteria are a good agreement if the BA ratio <0.1, a moderate agreement if 0.1 < BA ratio < 0.2 and a poor agreement if the BA ratio >0.2 [25].

ECG and PPG were measured with the instruments, and they were measured at the same time. We calculated the PCC with the time shift, and the maximum PCC can be considered as the synchronization between PPG and ECG.

### 2.4. Two Acupuncture Points Were Chosen in This Study

**Figure 2:**
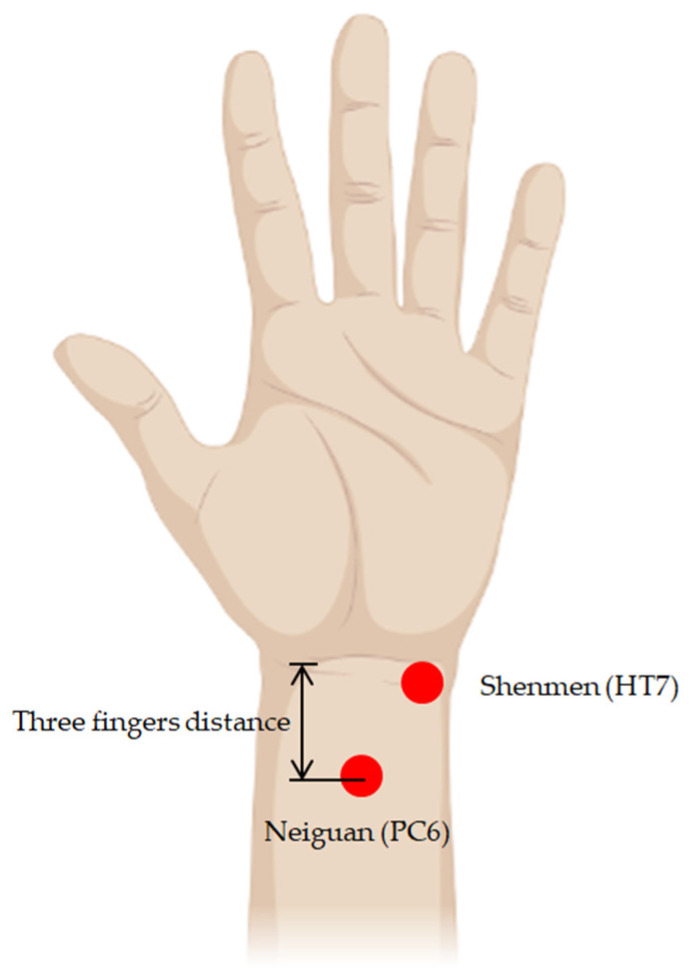
The positions of the acupuncture points of Neiguan (PC6) and Shenmen (HT7). Illustration created with BioRender.com (accessed on 30 October 2022).

Acupuncture is an important therapy method in traditional Chinese medicine (TCM). The first document that unequivocally described an organized system of diagnosis and treatment is *The Yellow Emperor’s Classic of Internal Medicine*, dating from about 100 BCE [29]. In 1989, the WHO Scientific Group adopted a Standard International Acupuncture Nomenclature. This standard nomenclature facilitates the teaching, research and clinical practice of acupuncture and the exchange in information globally [30]. In addition, a brief explanation of 361 classical acupuncture point names and their multilingual comparative list are explanted. In addition, the name of the meridian alphabetic code is revised. For example, the heart meridian is “HT”, and the pericardium meridian is “PC” [31]. In addition, eight extra meridians and extra points are listed [32] in the WHO public edition. In TCM theory, the Neiguan (PC6) acupoint belongs to the sixth acupoint of the pericardium meridian (PC). Acupuncture on the PC6 point is used to calm the mind, soothe the nerves, regulate qi and relieve pain [33]. In addition, acupressure at Neiguan (PC6) affects nausea, vomiting and comfort level in the postoperative period [34]. Furthermore, Shenmen (HT7) is a door for the mind [31]. It is the seventh acupoint of the heart meridian (HT), which means “Spirit Gate”, a gate to access our spirit. Acupuncture on Shenmen (HT7) can increase heart energy, is beneficial for insomnia, epilepsy and psychosis, and is the main point for heart disease, such as palpitation, cardiac pain, tachycardia, arrhythmia, etc. [35]. Because PC6 and HT7 are on the wrist, it is easy to perform PBM with the wearable device. We think there may be a similar effect on the other acupoints of the same meridian, for example, PC1-PC9 on the pericardium channel and HT1-HT9 on the heart meridian. The positions of Neiguan (PC6) and Shenmen (HT7) are shown in Figure 2.

Physiological status and psychological status are associated with brain waves, and there are mainly five kinds of brain waves. The rhythm names and the frequency bands are delta (0.5–3.5 Hz), theta (4–7 Hz), alpha (8–13 Hz), beta (13–30 Hz) and gamma (32–100 Hz). Alpha rhythm is the dominant brainwave in normal adults who are awake and relaxed with closed eyes, decreasing under the eyes-open or mentally active condition [36,37]. Gamma rhythms are associated with brain network activity and cognitive phenomena such as memory, attention, object recognition and perceptual processing [38]. The amplitude of the gamma rhythm can be increased by meditation [39] or neural stimulation, with the 40 Hz point being of particular significance [40,41]. PBM can be classified as two types, continuous wave (CW) and pulse wave (PW). PW-PBM can facilitate dermal wound healing in immunosuppressed rats [42]. In our previous studies, the low power of PW-PBM can cause significant changes in the intensity of the subject’s brain waves [20,21,22]. Therefore, the PBM effect may be enhanced by matching the pulse rate with the specific brain rhythm on the acupoints. In this pilot study, we stimulate the acupoints with different pulse rates of PW-PBM. One test is that PC6 acupuncture was stimulated with a 10 Hz pulse wave (during the alpha rhythm 8–13 Hz) to calm the hyperactive state. Another test is that HT7 acupuncture was stimulated with a 40 Hz pulse wave during the 32–100 Hz gamma rhythm to increase the hypoactive state. That means the LA with specific frequencies was performed, 10 Hz for Neiguan (PC6) and 40 Hz for Shenmen (HT7).

### 2.5. Participant and Test Procedure

**Figure 3:**
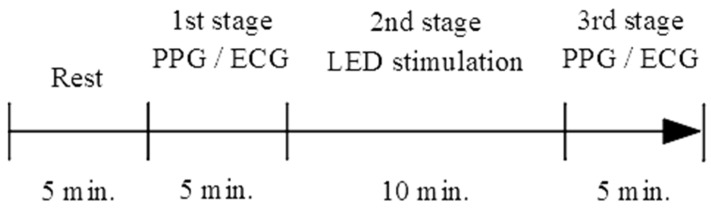
The test procedure with LED stimulation and PPG and ECG record.

Two subjects participated in the pilot test. The informed consents were signed by the subjects before this pilot study. One is a 57-year-old man, and another is a 31-year-old woman. They sat on a chair and kept awake during the test. At first, the subject was settled down for five minutes to stabilize the physiological state. There are three stages in the test procedure. The first stage is PPG and ECG measurement for five minutes, the second is LED stimulation for ten minutes and the third is PPG and ECG measurement for five minutes. The test procedure is shown in Figure 3.

## 3. Results

### 3.1. Signal Analysis of PPG and ECG Data

**Figure 4:**
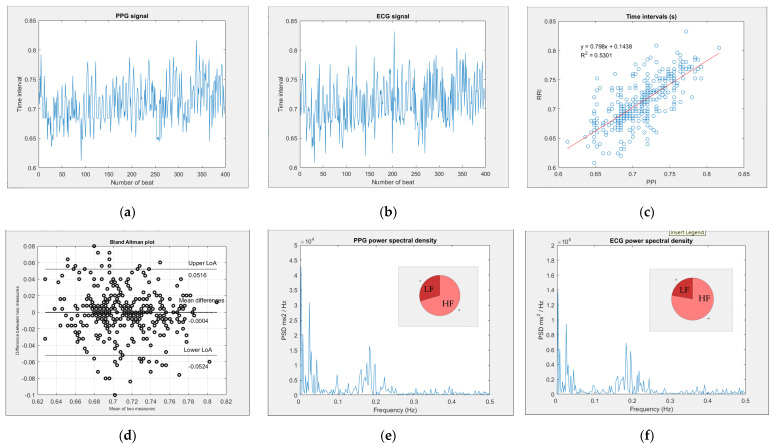
The analysis before LA with PPG and ECG measurements. (**a**) PPI plot in PPG signal. (**b**) RRI plot in ECG signal. (**c**) The time interval between PPI and RRI. (**d**) The Bland–Altman plot between PPI and RRI. (**e**) The power spectrum of PPG. (**f**) The power spectrum of ECG.

Regarding the analysis of PPG, the peak-peak intervals (PPI) of the PPG signal were calculated first, and then HRV metrics were executed in the time and frequency domains. Regarding the analysis of ECG, the R-R interval (RRI) of ECG, the time elapsed between two successive R-waves of the QRS signal, is from the “CheckMyHeart” device, then HRV metrics were executed in the time and frequency domains. Figure 4 is an example showing the analysis of the subject with PPG and ECG measurements before LA. The PPI chart of the PPG signal is shown in Figure 4a. The RRI chart of the ECG signal is shown in Figure 4b. The time intervals between PPI and RRI are shown in Figure 4c: the PCC value (R) is 0.7281 and the LCCC value is 0.5279. The Bland–Altman plot between PPI and RRI is shown in Figure 4d, and the BA ratio is 0.0732. In addition, the power spectrum of PPG is shown in Figure 4e, and the power spectrum of ECG is shown in Figure 4f.

### 3.2. The Effect of LED Stimulation on Two Subjects

**Figure 5:**
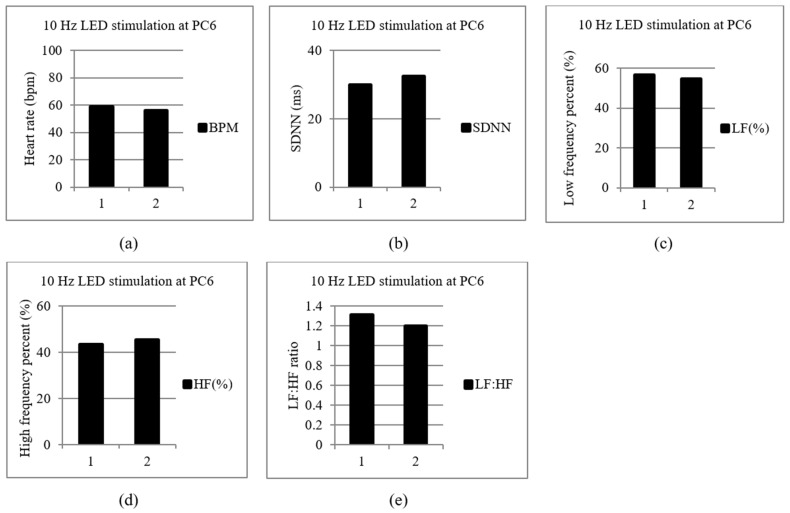
A 57-year-old male subject was stimulated with 10 Hz LA at Neiguan (PC6). The 1st time is before LA, and the 2nd time is after LA. (**a**) Beat per minutes (BPM). (**b**) The standard deviation (SDNN). (**c**) Low-frequency percent (LF (%)). (**d**) High-frequency percent (HF (%)). (**e**) LF: HF ratio.

In this study, two participants were recruited in the pilot test to observe the variety of ANS statuses by stimulating different acupoints with LA. Table 1 is the reference criterion which is not a standard. A 57-year-old male was accepted 10 Hz LA at Neiguan (PC6) due to his low SDNN and slightly higher LF (%) before LA, and the test results are shown in Figure 5. The beats per minute (BPM), SDNN, LF (%), HF (%) and LF: HF ratio are shown in Figure 5a–e, respectively. The subject’s SDNN and HF (%) were slightly increased and LF (%) was decreased slightly after 10 Hz LA.

**Figure 6:**
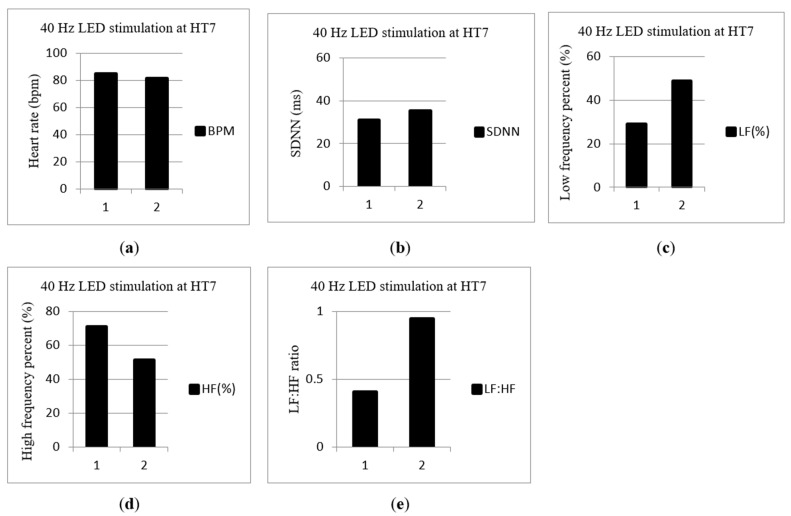
A 31-year-old female subject was stimulated with 40 Hz LA at Shenmen (HT7). The 1st time is before LA, and the 2nd time is after LA. (**a**) Beat per minutes (BPM). (**b**) The standard deviation (SDNN). (**c**) Low-frequency percent (LF (%)). (**d**) High-frequency percent (HF (%)). (**e**) LF: HF ratio.

In addition, a 31-year-old female whose SDNN and LF (%) were lower before LA accepted 40 Hz LA at Shenmen (HT7), and the test results are shown in Figure 6. The BPM, SDNN, LF (%), HF (%) and LF: HF ratio are shown in Figure 6a–e, respectively. The subject’s SDNN and LF (%) were increased after 40 Hz LA.

## 4. Discussion

The ANS plays an essential role in life support, such as breathing, blood circulation, metabolism, digestion, secretion, body temperature, reproduction, etc., to maintain the functional stability of the internal environment and keep the persistence of life. The ANS includes the SNS and the PNS, and both have antagonistic and control effects on internal organs and blood vessels [43]. The analysis of the HRV has been used for clinical applications. For example, the estimated 5-year mortality of diabetic autonomic neuropathy (DAN) is approximately 50% [44]. HRV can be used to assess DAN [45]. Thus, if we can frequently measure HRV and regulate the ANS with LA immediately, the mortality of DAN could be reduced. In this work, we aimed to develop a system not only to monitor the ANS status, but also can regulate ANS status immediately. HRV metrics were calculated from the PPG signal, and ANS status were analyzed from HRV metrics. In addition, the agreements between PPG and ECG were compared. In our previous studies, PBM has the influence to affect the ANS status [19], and similar effects were also observed in this study.

For the ECG signal, R points of the QRS complex waves were used for the time interval of the heartbeat. For the PPG signal, the peaks were used for the time interval of the heartbeat. If PPG and ECG have prominent peaks and the contour, the PPI and RRI can be calculated correctly. However, if there were motion-generated artifacts or weak reflected signals in the PPG signal, it could obtain the wrong PPI. There is a similar situation in the ECG signal. If the arrhythmias are present in the heart, it can cause abnormal RRI. It is worth noting that if the peaks from PPG and the peaks from ECG are not chosen correctly, it may cause the deviation of time intervals, and HRV metrics between PPG and ECG will be different. GARY G [46] found that even one beat can affect the HRV and power PSD.

We used three methods to assess the agreement between PPI and RRI. In Figure 4, the Pearson correlation coefficient is 0.7281, the value is between 0.7 and 0.9 and the performance is a high positive correlation. For the Bland–Altman analysis, the BA ratio is 0.0732 and the value is lower than 0.1, so it is a good performance. However, Lin’s Concordance Correlation Coefficient is 0.5279, which is lower than 0.9, and therefore it is a poor performance. It is worth noticing that a poor agreement between PPI and RRI does not mean that the signal obtained from the PPG sensor lacks quality. It can still monitor changes in HRV metrics with poor concordance. This only tells us that these HRV metrics cannot be used interchangeably with the ones obtained from the ECG signal [25]. PPG can still be used for monitoring the changes in SDNN, LF (%) and HF (%).

Autonomic drugs can inhibit or activate the function of the parasympathetic and sympathetic nervous systems. These drugs treat conditions such as glaucoma, asthma, urological disorders, gastrointestinal disorders and cardiopulmonary disorders [47]. The ANS can be adjusted by acupuncture, and the subjects were in fatigue and non-fatigue states. PNS and the SNS were activated by acupuncture needling at Hegu (LI4) and Neiguan (PC6) [48]. In addition, some non-invasive autonomic neuromodulation techniques for treating cardiovascular diseases include transcutaneous auricular vagus nerve stimulation, electromagnetic field stimulation, ultrasound stimulation, optogenetics for stimulation and transcutaneous cervical nerve stimulation [49]. In this study, photobiostimulation was used. The wavelength 850 nm of an LED is infrared light, which is suitable for deeper skin penetration to the hypodermis [50]. This system uses LED to radiate the acupoints to affect the ANS, since low-power infrared LED light is invisible and no heat is emitted. LA has the advantages of safety and no side effects. On the other hand, the optical profile or radiation pattern of the light source is a parameter that affects the distribution of light in our tissue [51], thus affecting the efficiency of PBM. Dosage is another parameter that affects the effect of LA on adjusting the ANS [52]. The ANS may be adjusted by changing the dose on the same acupoint.

In case one, in Figure 5, regarding the 10 Hz stimulation at Neiguan (PC6), SDNN and HF (%) were increased; LF (%) was decreased after LA. An increase in HF (%) means the PNS was activated. In this case, the subject was treated with LA at the Neiguan point, the PNS was excited, and the SNS was inhibited. The test result matches our previous study [19]. In case two, in Figure 6, the subject’s SDNN and LF (%) were increased after LA at Shenmen (HT7) with 40 Hz. SDNN and LF (%) were increased; HF (%) was decreased after LA. Increased LF (%) means that the SNS was excited and the PNS was inhibited. In this study, two subjects participated in the pilot study to confirm the functionality and feasibility of the prototype. However, in the future, a larger group of subjects needs to be studied to confirm the ANS effect at the specific acupoint and the pulse rate.

**Figure 7:**
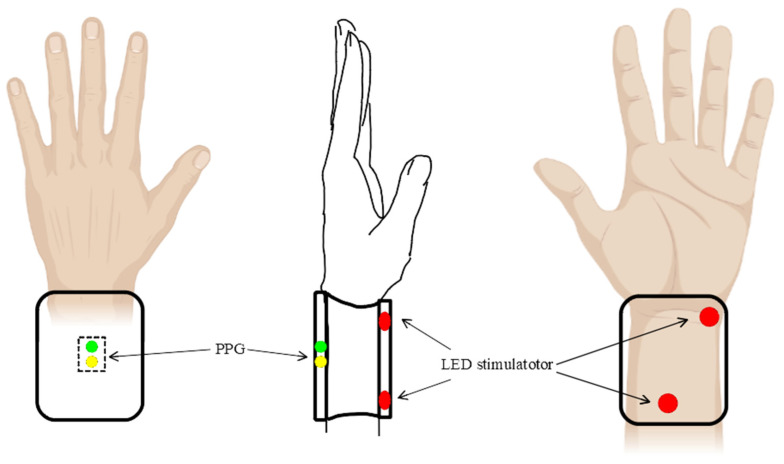
A conceptual wearable device with PPG record and LA. Illustration created with BioRender.com (accessed on 30 October 2022).

The proposed system can be developed for miniaturization and wireless communication in the future. The size of the system can be miniaturized as a wearable device, such as a watch-integrated LA and PPG monitor, as shown in Figure 7. This system can be applied to many fields. For example, the relative mortality risk is 5.3 times higher in the group with HRV of less than 50 ms than in the group with HRV of more than 100 ms after acute myocardial infarction (AMI) [24]. HRV is highly age and gender dependent. Younger people have higher SDNN than older people. Men have slightly higher SDNN than women [53]. In this study, the HRV (SDNN) values of the two cases increased after LA. The implication of this study is that the system we proposed is not just suitable for normal subjects but also helpful for patients with DAN or AMI.

## 5. Conclusions

This study proposed a real-time system integrated with a PPG sensor and LA to measure HRV and regulate the ANS. A comparison of PPG and ECG was conducted, and the consistency of these two methods was discussed. The pilot tests were conducted with this proposed system. With 10 Hz LA at the Neiguan (PC6) acupoint, SDNN and HF (%) of the subject were increased. On the contrary, when stimulating at the Shenmen (HT7) acupoint with 40 Hz LA, SDNN and LF (%) were increased. The preliminary tests showed that this proposed system has the potential application to measure and affect the ANS status in real-time. Clinical trials approved by the Ethical Committee will be conducted to confirm the effect in the future.

## Figures and Tables

**Table 1:** Referred normal values of standard measure of HRV [2].

Variable	Normal Values (Mean ± SD)	Note
Time domain analysis of normal 24 h		
SDNN (ms)	141 ± 39	Standard deviation of normal-to-normal intervals
Frequency domain analysis of supine 5 min recording		
LF (nu)	54 ± 4	LF power in normalized units LF/(total power − VLF) × 100
HF (nu)	29 ± 3	HF power in normalized units HF/(total power − VLF) × 100
LF/HF	1.5–2.0	Ratio LF (ms^2^)/ HF (ms^2^)

## Data Availability

The data used to support the findings of this study are included within the article.
